# Schiff Base Switch II Precedes the Retinal Thermal Isomerization in the Photocycle of Bacteriorhodopsin

**DOI:** 10.1371/journal.pone.0069882

**Published:** 2013-07-29

**Authors:** Ting Wang, Marc T. Facciotti, Yong Duan

**Affiliations:** 1 UC Davis Genome Center, University of California Davis, Davis, California, United States of America; 2 Department of Biomedical Engineering, University of California Davis, Davis, California, United States of America; University of Akron, United States of America

## Abstract

In bacteriorhodopsin, the order of molecular events that control the cytoplasmic or extracellular accessibility of the Schiff bases (SB) are not well understood. We use molecular dynamics simulations to study a process involved in the second accessibility switch of SB that occurs after its reprotonation in the N intermediate of the photocycle. We find that once protonated, the SB C15 = NZ bond switches from a cytoplasmic facing (*13-cis*, *15-anti*) configuration to an extracellular facing (13-*cis, 15-syn*) configuration on the pico to nanosecond timescale. Significantly, rotation about the retinal’s C13 = C14 double bond is not observed. The dynamics of the isomeric state transitions of the protonated SB are strongly influenced by the surrounding charges and dielectric effects of other buried ions, particularly D96 and D212. Our simulations indicate that the thermal isomerization of retinal from *13-cis* back to *all-trans* likely occurs independently from and after the SB C15 = NZ rotation in the N-to-O transition.

## Introduction

Bacteriorhodopsin (BR) functions as a light-activated proton pump [1], [2]. Its catalytic activity is dependent on a retinal chromophore that is covalently bound to residue K216 via a Schiff base (SB) C15 = NZ linkage. In the light-adapted ground state, the retinal’s polyene chain rests in the *all-trans* configuration and the SB is protonated and in a *15-anti* configuration. In this state, the SB nitrogen points towards the extracellular (EC) side of the protein. Absorption of an actinic photon isomerizes the retinal’s C13 = C14 double bond to a *13-cis* configuration and together with molecular changes in the protein moiety triggers a proton transfer from the SB to D85 and a coupled release of a proton to the extracellular side. These molecular events characterize the first half of BR’s photocycle, and are associated with spectroscopically identifiable intermediate states termed K, L, and M. In the second half of the photocycle, the SB is reprotonated by D96, a residue located at the cytoplasmic (CP) side. The reprotonation of D96, deprotonation of D85, and thermal isomerization of the retinal to an *all-trans* configuration restore the initial ground state. These last steps occur during the photo-intermediate states N and O. The net effect of the photocycle is the transport of a proton out of the cell (see review [3]–[7] for the photocycle of BR).

Implicit in the vectorial pumping mechanism is a requirement for the SB nitrogen to switch its accessibility between the EC and CP sides of the protein. In the first half of the photocycle the SB nitrogen switches its accessibility from the EC to CP sides of the protein (we term this switch I) while in the second half it reverses its accessibility back to the EC side (we term this switch II). The accessibility switch is so tightly coupled to the proper operation of BR that its molecular underpinnings may even be conserved in sensory rhodopsins as critical mechanistic components of the switch between photophobic and phototactic signaling states [8]. Given this centrality of function, the accessibility switch has been extensively explored in structural [9]–[12], spectroscopic [13]–[17], and computational studies [18]–[21]. Hypotheses regarding the molecular determinants of the accessibility switches have implicated the retinal/SB configuration [9], [16], [18]–[20], [22], changes in protein structure [13], [14], [23], [24], or a combination of multiple factors including water molecules [15], [25]–[29] as contributors to the switch function.

The contributions of the retinal and SB to the switch mechanism are mostly related to bond rotations about the C13 = C14, C14–C15, and C15 = NZ bonds and their ability to orient the SB nitrogen between CP and EC pointing states. X-ray crystal structures of early photointermediate states have shown that the SB transitions from an EC (13-*cis, 15-syn*) to a CP (*13-cis*, *15-anti*) pointing configuration after its deprotonation and the photo-isomerization of the retinal around the C13 = C14 bond [10], [11], [30]–[32]. This configurational change is temporally linked to switch I during a transition between early and late sub-states of the M intermediate, a portion of the photocycle that also experiences a large irreversible drop in free energy [33]. Despite the abundance of structural data describing the steps preceding and immediately after switch I, a detailed molecular mechanism explaining this process remains elusive. Even describing the bond rotations that must occur to reorient the SB is challenging owing to the unfavorable energetics associated with rotation about the C15 = NZ double bond after the SB has deprotonated [34]. In addition, the numerous complementary changes in protein structure that develop between the ground state and the flip of switch I must also be taken into consideration.

**Figure 1:**
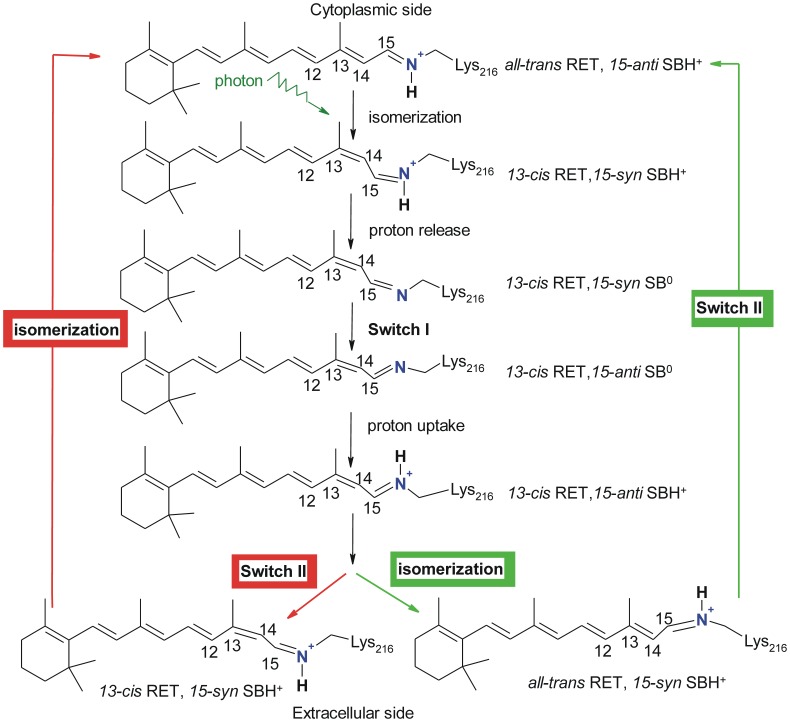
Schematic view of the photocycle of bacteriorhodopsin. The accessibility and orientation of the SB nitrogen atom undergoes two switches. In switch I, de-protonated SB (SB^0^) changes its orientation from extracellular to cytoplasmic; in switch II, re-protonated SB (SBH^+^) changes its orientation from cytoplasmic to extracellular. We aim to understand whether switch II occurs before the thermal isomerization of retinal (the red route) or after (the green route).

The molecular regulation of the switch from the CP-accessible state back to the EC-accessible switch (switch II) remains equally mysterious and, by comparison to switch I, understudied. One key challenge to studying switch II is that unlike switch I, there are very few high-resolution structures available for intermediates occurring after the M state to draw from. The fact that no N or O intermediate structures have been solved with the wild type protein further compounds the problem. In this work we investigate two potentially critical molecular reactions involving configurational switches in the retinal and SB: the isomerization of the C15 = NZ bond of the re-protonated SB and the thermal isomerization of the retinal C13 = C14 bond (**Figure 1**). We also describe the influence of the protein state on the configurational kinetics of the retinal and SB by simulating the effects of different protonation states on critical amino acids.

Since the following analysis and discussion focus heavily on describing the isomerization of retinal and SB bonds it is instructive to note that the change of the isomeric state of the retinal C13 = C14 bond will change the isomeric state of the C15 = NZ bond, even if no rotation happens around the C15 = NZ bond. For example, the configuration of the retinal in the light-adapted ground state is termed *all-trans*, *15-anti*. After photo-isomerization of the retinal C13 = C14 bond, the configuration will be termed *13-cis* and *15-syn* even though no rotation has occurred around the C15 = NZ bond and the SB nitrogen still points towards the EC side. Similarly, retinal’s isomerization from *13-cis* to *all-trans* will convert a *15-anti* C15 = NZ bond to *15-syn* even though no rotation around the C15 = NZ bond has occurred and the SB nitrogen still points to the CP side. In other words, only in the case that the retinal C13 = C14 bond remains its isomeric state, a change of the isomeric state of the SB C15 = NZ bond indicates a change of the SB nitrogen accessibility (switch I or switch II). For reference, **Figure 1** shows the configurational state of the retinal and the SB in each state of the photocycle.

Experimental data show that during the wild-type photocycle the retinal is *13-cis* in the N intermediate and that it re-isomerizes back to *all-trans* only in the O state on a millisecond time scale [35], [36]. The activation energy barrier was computed to be ∼14 kcal/mol when D85 was modeled in a protonated form, mimicking its state during the N-to-O transition [21]. By contrast, there is no experimental or computational data about the kinetics or energetics of switch II and it is not clear whether switch II occurs before or after the retinal’s *cis* to *trans* isomerization. We do know, however, that several important residues and molecular transformations that happen between the M and O intermediates might affect the order of events in switch II. For instance we know that residue replacements in the retinal cavity can have a significant impact on retinal isomerization states and rates of configurational transitions. The anion D212 is known to be particularly influential [37]–[40]. In addition, we know that D96 cycles between protonated and deprotonated states between the M and O intermediates. This influences the local electrostatics, changes the protonation state of the SB and, as we have recently found, when D96 is deprotonated the cytoplasmic proton uptake pathway opens, forming a water channel that connects the cytoplasmic bulk to the SB [41].

To characterize the behavior of the retinal and SB during switch II and the effects of the key ionizable residues D212 and D96, we started by investigating the high-resolution x-ray structure of an N’ photointermediate [42] (PDBid 1P8U) that was determined by photoconverting crystals of the V49A mutant at pH 5.6. Under these conditions the SB is assumed to be protonated [5], [42], [43] and the retinal and SB is in a *13-cis*, *15-anti* configuration. We also investigated three M state x-ray crystal structures [10]–[12](PDBID 1KG8, 1F4Z and 1C8S). In each of the M structures, the retinal and SB is in a *13-cis*, *15-anti* configuration, but unlike the N’ intermediate the SB is presumed to be deprotonated. Starting with the atomic coordinates for each of the four structures, we performed molecular dynamics (MD) simulations to investigate the dynamics of the isomeric state of the SB C15 = NZ double bond in response to changes in the protonation states of the SB, D96, and D212.

We observed that a protonated CP-pointing SB switches quickly to an EC-pointing configuration and forms a hydrogen bond with the anionic D212. In all simulations the retinal remained in the *13-cis* configuration. By contrast, when the SB was modeled in an unprotonated state, it remained pointing towards the CP. We also found that the protonation states of D96 and D212 have profound effects on the isomeric state preference of the protonated SB. Both deprotonation of D96 and protonation of D212 increase the population of the CP-pointing configuration and delay the switch toward the EC side. Based these observations, we propose a revised proton isomerization-transfer-switch model.

## Materials and Methods

### BR Structures and Modeling

The N’ intermediate state x-ray crystal structure (PDB code 1P8U) [42], the M1 intermediate state structure (PDB code 1KG8) [10], the M2 intermediate structure (PDB code 1F4Z) [11] and the Mn intermediate state structure (PDB code 1C8S) [12] were used in our simulations. The detail of the properties of these structures is listed in **Table S1**.

None of the crystal structures has the complete sequence of 248 residues encoded in the *bop* gene. The first four N-terminal residues are missing in all structures, as well as the last 17 C-terminal residues. In addition, the EF loop is missing in all the structures. For each bR model, we included the protein, the retinal molecule and all crystal water molecules. The lipid and organic solvent molecules solved together with the proteins in the crystal structures were excluded. The protein residues not solved in the crystal structures were not modeled in except for the EF loop, which we modeled in by using the loop modeling web server [44]. In addition, the D96N mutation in the Mn structure was mutated back to the wild type.

### Protonation States of the Asp and Glu Residues

As we were studying the events occurring after the Schiff base has released a proton, D85, the proton acceptor of the Schiff base was modeled in a protonated state in all simulations. D115, a residue located beside the proton transport pathway was also modeled in the protonated state so that it could form a hydrogen bond with T90. The protonation states of D96 and D212 were varied depending on the focus of the simulations. All other Asp and Glu residues were modeled in deprotonated states in all simulations.

### Protonation States of the Schiff Base

In the simulations that mimicked the states in which the Schiff base had accepted a proton from D96 (rising and decay of the N state) the Schiff base was protonated. In the simulations that mimicked the M state the Schiff base was deprotonated.

### Schiff Base Alone in Solution

The *13-cis* retinal, protonated Schiff base and the connecting residue K216 was extracted from the 1P8U structure. The N and C-termini of K216 were neutralized by adding the ACE and NME residues, respectively. This modeled molecule was then solvated in a water box with a boundary of 12 Å and ion concentration of 200 mM.

### Modeling of bR in Solvated Membrane Environment

First, we used the REDUCE program [45] to add hydrogen atoms into each bR structure. We then embedded each structure into a lipid bilayer. The palmitoyloleoyl-phosphatidylcholine (POPC) lipid molecule was used and the VMD1.8.6 program [46] was used to generate a patch of POPC lipid membrane with a dimension of ca. 100 Å ×100Å. Each bR model was then aligned with the axes of the membrane and the center of mass of protein was translated to the center of mass of the membrane by using the combine.tcl script available on the web page of the VMD program. Lipid molecules within 1.5Å of the protein were removed. Depending on the individual bR structure, the number of the lipid molecules in each model was between 119 and 122.

Next, each bR model embedded in the membrane was then solvated in water in the Z-direction, i.e. at the intracellular and extracellular sides parallel to the membrane plane. This was carried out by using the tLeap module in the AMBER program [47] and an in-house script. The salt concentration was set to 200 mM by adding chloride and sodium ions. This salt concentration is lower than the physiological salt concentration for *H. salinarum* (4 M) [48], but it is typical and practical for MD simulations. Indeed, Pezeshki et al. have even recently shown that an ionic composition of 1M may be a practical upper bound for accurately simulating the effects of ions in solution [49]. In addition, for practical reasons (limiting simulation size), in our most recent publication [41] we formally investigated whether there was any difference between the use of 200mM or 1M on the behavior of BR systems - we found nothing substantive. Depending on the individual bR structure, the total numbers of atoms in the simulations presented in this manuscript are between 43,168 and 43,546.

### MD Simulations

Each of the solvated bR-membrane systems was first subject to a two-stage energy minimization. In the first stage of 2000 steps, bR was restrained to its crystallographic positions by a harmonic potential with a force constant of 32 kcal/(mol·Å^2^) while all other atoms were unrestrained. In the second stage of 2000 steps, no restraint was applied. In both stages, the steepest decent method was used for the first 10 steps and the conjugate gradient method was switched on for the rest of the steps.

After energy minimization, the whole system was subjected to a three-stage equilibration MD simulation. In the first stage, the system was gradually heated from 10K to 300K in 50 ps and with constant volume while bR, retinal and the head groups of the lipid molecules were restrained by a force constant of 32 kcal/(mol·Å^2^). The restraints on the head-groups were used to prevent the tails from interdigitating, which would otherwise result in the reduction of the bilayer thickness. In the second stage, the restraints on the protein and the lipid molecules remained but the system was simulated at constant temperature of 300K and constant pressure of 1.0 atm for 500ps. In the third stage, the restraints on the lipid molecules were removed and the system was simulated for 500ps with the restraints remaining on the protein. After the equilibration simulation, the MD simulation continued with all atoms free. The unrestrained MD simulation was the production run used for our analysis. We performed 48 simulations with a total length of 5.032 µs. The detail of the simulations was summarized in Table S2.

Retinal, including the Schiff base and three terminal carbon atoms of Lys216 were treated as a single unit and the parameters were obtained by using the Antechamber module and the general force field (GAFF) in the AMBER program. The remaining part of Lys216 was parameterized based on residue Ala, connecting the Schiff base part by a single bond. Here we would like to note that the parameters involving the NZ atom in the Schiff base, in particular the dihedral angle parameters of the C15 = NZ bond, are different when the Schiff base is protonation and deprotonated. The parameters of the POPC lipid molecules were taken from our previous work [50], which were also derived by using the Antechamber module and GAFF force field in the AMBER program. The parameters of protein residues were assigned based on the AMBER ff03 force field [51]. For water molecules, the TIP3P model was used.

The MD simulations were carried out by using the GPU-CUDA version of the AMBER11 program. The Particle Mesh Ewald (PME) method [52] was used for long-range electrostatic interactions with the default parameters. The bonds involving hydrogen atoms were constrained by using the SHAKE algorithm. The time step was 1 fs in the equilibration simulation and 2 fs in the production run, and the non-bonded interactions were updated every 10 time steps. The simulation trajectories were saved every 10 ps.

### Trajectory Analysis

#### SB accessibility

The accessibility of the SB nitrogen atom is determined by the isomeric state of the C15 = NZ double bond. With the retinal C13 = C14 double bond in the *cis* configuration (conventionally denoted *13-cis*), if the C15 = NZ double bond is *trans* (conventionally denoted *15-anti*), the SB nitrogen points to the CP side; if it is *cis* (conventionally denoted *15-syn*) then pointing to the EC side. For each of the 48 MD simulation trajectories, we computed the dihedral angle C14-C15 = NZ-CE. We define that a C14-C15 = NZ-CE dihedral angle in the range of from −60 to 60 degree indicates a *15-syn* configuration and that in the range of from 120 to 180 degree or from −180 to −120 degree indicates a *15-anti* configuration. Similarly we also computed the C12-C13 = C14-C15 dihedral angle in each simulation.

#### Hydration of the cytoplasmic proton uptake pathway

We previously found that when D96 is deprotonated, a water channel forms along the cytoplasmic proton uptake pathway, connecting the SB site and the cytoplasm. The hydration of the pathway was characterized by the number of water molecules that accessed the cavity between D96 and K216. This cavity is approximated by a sphere that is centered at the midpoint of the line connecting the D96:CA atom and the K216:CA atom. The radius of the sphere is the half distance between those two atoms minus 1 Å. As a reference, in the four crystal structures used in this work, the distances between the D96:CA atom and the K216:CA atom are between 11.63 to 11.94 Å and the numbers of crystal water molecules in the cavities are between 2 and 4 (**Table S1**). It is worth noting that WAT501 in the M2 state (1f4z) and WAT504 in the N’ state (1p8u) were excluded by this definition. For each simulation, we computed the total number of the different water molecules that accessed the D96-K216 cavity as well as the frame-averaged number of the water molecules. The water channels formed in simulations were visualized and characterized by using the MOLE web server [53].

## Results

We performed a series of molecular dynamics (MD) simulations of BR embedded in a solvated lipid bilayer. The first set of the simulations were based on the x-ray crystal structure in the N’ state (PDBid 1P8U). Additional simulations were performed based on three M state x-ray crystal structures (PDBids 1KG8, 1F4Z and 1C8S) (**Table S1**). In each crystal structure, the retinal and SB is in a *13-cis*, *15-anti* configuration, indicating a CP-pointing SB. The isomeric state of the C15 = NZ double bond or the orientation of the SB nitrogen atom was the focus of our investigation. Both protonated and unprotonated SBs were modeled and simulated. The protonation states of D96 and D212 were also varied to examine their effects on the isomerization of the SB. In all simulations, D85 was protonated to correspond to the photointermediates after the proton release from the SB to D85 that are of relevance to Switch II, i.e. the M, N, and O states. The simulations ranged in length from 45.74 ns to 184.45 ns, resulting in a total of 5.0 µs (**Table S2**).

### Simulations Based on the N’–state Structure

#### Simulations with an unprotonated SB

**Figure 2:**
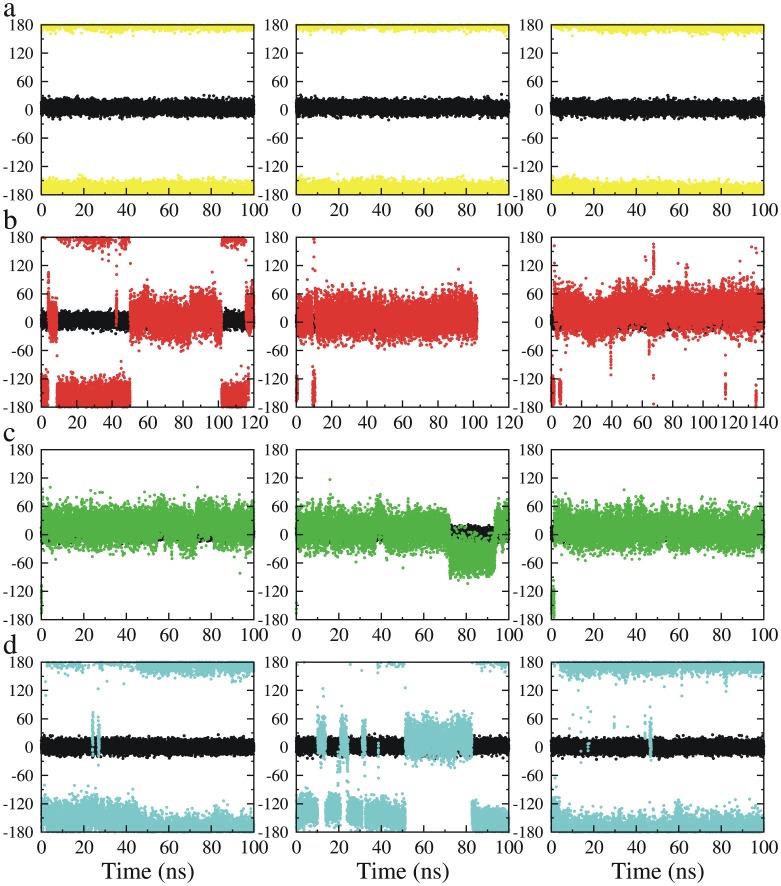
Time development of the dihedral angles C14-C15 = NZ-CE (color symbols) and C12-C13 = C14-C15 (black symbols) in the simulations starting from the N’ structure (1P8U). a) The SB was un-protonated and D96 was protonated, mimicking the M state; b) The SB was protonated and D96 was deprotonated, mimicking the rising of the N state; c) The SB was protonated and D96 was protonated, mimicking the decay of the N state; d) The SB, D96 and D212 were protonated. The black symbols in each of the plots depict the isomeric state of the retinal C13 = C14 double bond, which remained its initial *13-cis* configuration in all simulations.

Three triplicate simulations were performed in which the SB was modeled in an unprotonated state while D96 was modeled in a protonated state and D212 was modeled in a deprotonated state. This combination of protonation states correspond to that found in the wild-type M state, well before switch II should occur. In each of the 100-ns simulations, the SB remained its *15-anti* configuration. The retinal also remained its *13-cis* configuration throughout the simulations (**Figure 2a**).

#### Protonated SB dynamics with a protonated or deprotonated D96

In the native photocycle, switch II occurs after the SB is reprotonated by D96 during the N intermediate state. As the re-protonation of D96 is a rate-limiting step in the N-to-O transition [54], we thus modeled two sub-states of the reprotonated SB: one with deprotonated D96, mimicking the rise of the N intermediate and the other with protonated D96 mimicking the decay of the N state. Both models were subject to three triplicate simulations.

**Figure 3:**
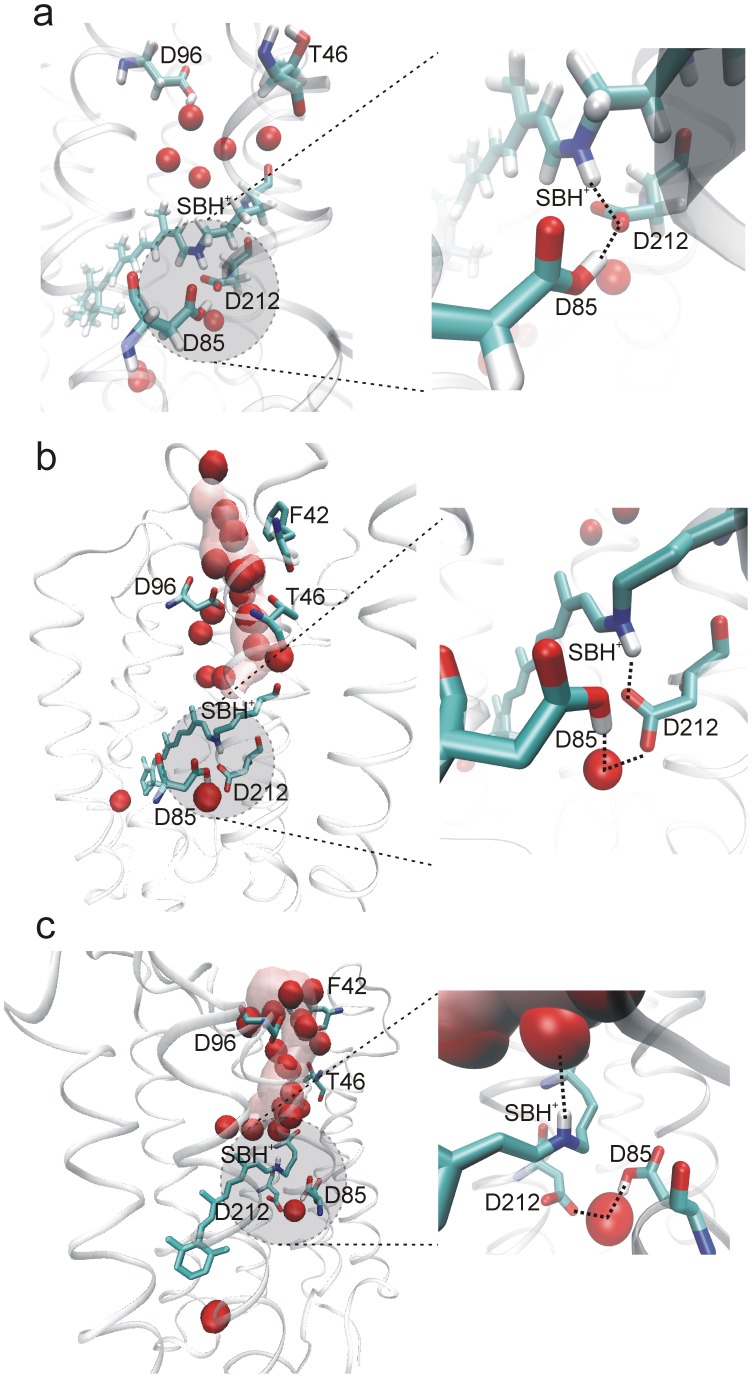
Structural snapshots of the protonated SB (SBH^+^) in different simulations. a) D96 was protonated. SBH^+^ was *15-syn*, pointing to the EC side and it formed a hydrogen bond with D212 which also formed a hydrogen bond with the protonated D85. One water molecule was in the close vicinity of both D212 and D85 at the EC side; b-c) D96 was deprotonated. The cytoplasmic proton uptake pathway opened and formed a water channel (transparent pink pipe). SBH^+^ was able to take two configurations: *15-syn*/EC-pointing as shown in b) and *15-anti*/CP-pointing as shown in c). When CP-pointing, SBH^+^ formed a hydrogen bond with one water molecule at the CP side and in the same time another water molecule bridged D85 and D212 at the EC side. For clarity, non-polar hydrogen atoms are not shown in b) and c).

In each of the three 100-ns simulations in which D96 was protonated, the protonated SB turned down quickly toward the EC side, exhibiting an isomeric state of *15-syn* during the rest of the simulations (**Figure 2b**). The length of time in which the protonated SB remained in its initial *15-anti* configuration was 210, 60, and 1410 ps, respectively in each of the three simulations. The EC-pointing SB nitrogen atom returned in close contact with the deprotonated D212 and protonated D85. Typically, the SB formed a hydrogen bond with D212, which also formed a hydrogen bond with D85. In addition, a water molecule was located in the close vicinity of D85 and D212 at the EC side, in a position similar to that of crystal water molecule WAT401 or WAT406 (**Figure 3a**).

**Figure 4:**
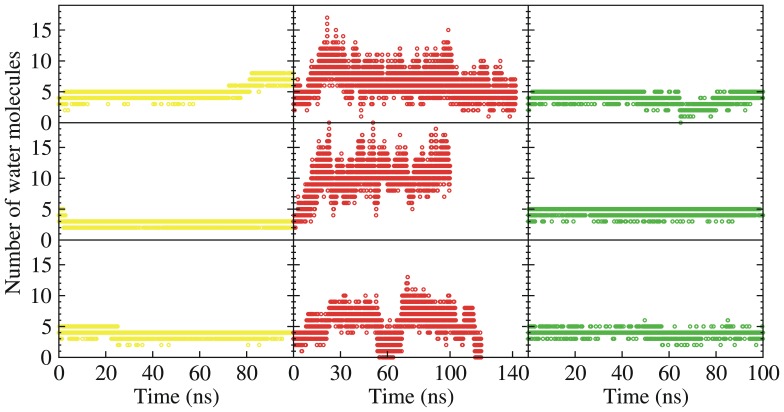
Time development of the number of the water molecules in the D96-K216 cavity. Left column: three simulations with un-protonated SB (SB^0^) and protonated D96; middle column: three simulations with protonated SB (SBH^+^) and deprotonated D96; right column: three simulations with protonated SB (SBH^+^) and protonated D96.see also **Figure S5**.

The SB behaved differently in the three simulations in which D96 was deprotonated. The initial *15-anti* configuration (CP-pointing) persisted longer than it had in the simulations with a protonated D96 and the SB exhibited spontaneous interconversion between the *15-syn-* and *15*-*anti-* isomeric states (**Figure 2c**). Consistent with our previous finding that the deprotonation of D96 unlatches the cytoplasmic proton uptake channel in BR [41], we observed that a water channel formed in all three simulations with a deprotonated D96, connecting the SB to the cytoplasm (**Figure 4**) and creating a hydrated local environment above the SB. When the protonated SB was CP-pointing (*15-anti*), the SB nitrogen atom formed a dynamic hydrogen bond with one of the water molecules that filled in the D96-K216 cavity (**Figure 3c**). On the extracellular side of the SB, we noted that one water molecule inserted between D212 and D85, extending the D212:OD2-D85:OD1 distance to around 6Å. The position of this water resembles that of the crystal water molecule WAT402 present in most of the x-ray structures in a state before the M state, including the ground (e.g. PDBids 1KGB [10] and 1C3W [55]), K (PDBid 1M0K [30]), L (PDBid 2NTW [31]) states.

To further examine how the hydration of the local environment of the SB affects its isomeric state, we performed a 110-ns simulation for a simplified model of the *13-cis* retinal, the protonated SB, and the connecting K216 in solution (see SI for details). As shown in **Figure S1**, the SB continuously interconverted between the *anti*- and *syn*- isomers, with the *anti*- isomer predominating. This suggests that when the both sides of the protonated SB are equally hydrated, the SB prefers the *anti*-isomer.

The behavior of the freely hydrated retinal-SB-lysine construct evokes the observations made on the SB in the simulations in which D96 was deprotonated. This suggests that the affinity of the SB for the CP-pointing configuration (*15-anti*) in the simulations in which D96 was deprotonated might be the consequence of the increased hydration of the CP side that accompanies this protonation state [41]. Meanwhile, the water molecule that inserts itself between D85 and D212 may also contribute to the stability of the CP-pointing configuration by decreasing the electrostatic attraction between both anionic residues and the protonated SB.

#### Protonated SB dynamics with a protonated D212

While changes in the hydration of the CP channel had clear effects on the dynamics of switch II, the preferred configuration of the protonated SB was always *15-syn* (EC-pointing) (**Figure 2a and 2b**). This suggested that an electrostatic attractor might be acting to “pull” the protonated SB towards the extracellular side of the protein. Given prior work, the most likely candidate would be the anion D212 [37], [38], [40]. To investigate whether D212 might be a contributing factor to the switch II kinetics observed earlier, we performed triplicate simulations in which the SB, D212, and D96 were protonated. As shown in **Figure 2d**, protonation of D212 almost abolished the EC-pointing configuration in two of the three 100-ns simulations where the protonated SB remained its initial CP-pointing configuration. In the third simulation, the EC-pointing and CP-pointing configurations alternated almost equally. In addition, careful analysis of simulation trajectories showed that the protonated D85 and D212, although neutralized, still had strong electrostatic interactions, as judged by the formation of hydrogen bonds between these residues and the EC-pointing protonated SB.

### Simulations Based on the M-state Crystal Structures

We wanted to address whether starting structural models affect the dynamics and kinetics of the SB switch II. We therefore performed similar simulations based on three crystal structures of M intermediate state (1KG8, 1F4Z and 1C8S). Just as in the simulations based on the N’ structure, the retinal and SB in the M intermediates began in the CP-pointing 13-*cis*, 15-*anti* configurations. A notable structural difference between the N’ structure and the three M structures is the lower number of the crystal water molecules in the D96-K216 cavity in the M structures. In the N’ structure, there are four such crystal water molecules while two, three, and two are present in 1KG8, 1F4Z, and 1C8S, respectively. In addition, these three M structures represent three slightly different sub-states. 1KG8 is an early M state of the wild type BR, denoted as the M1 state; 1F4Z is a late M state with an E204Q mutation, denoted as the M2 state; 1C8S is an accumulated M state with a D96N mutation, denoted as the M_N_ state.

**Figure 5:**
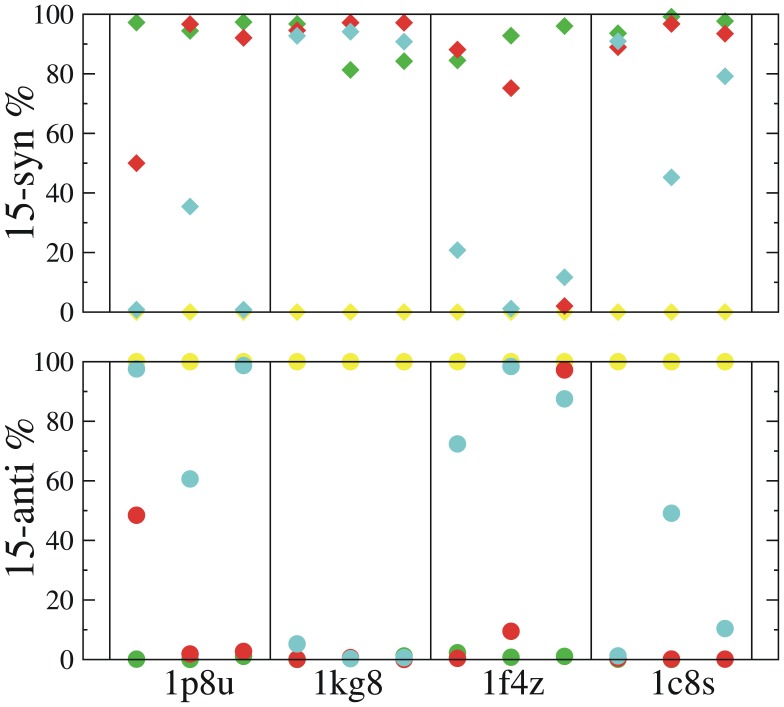
Percent occupancies of the *15-anti*/CP-pointing (lower panel) and *15-syn*/EC-pointing (upper panel) configurations of the SB C15 = NZ bond in each of 48 simulations based on crystal structure of 1p8u, 1kg8, 1f4z and 1c8s. Simulations starting from a same crystal structure are boxed in one column. Yellow symbols represent the simulations in which the SB is unprotonated and D96 is protonated; red symbols represent the simulations in which the SB is protonated and D96 is deprotonated; green symbols represent the simulations in which both the SB and D96 are protonated; cyan symbols represent the simulations in which the SB, D96 and D212 are protonated; For each of the protonation state variations, three runs are performed and they are represented by a same color but placed separately along the x-axis in a crystal structure box. See also Figures S2, S3 and S4.

Additional simulations with these three starting structures revealed a picture largely consistent with that discovered in the simulations of the N’ intermediate (**Figure 5** **and Figures S2–S4**). In all cases, the unprotonated SB remained CP-pointing. Meanwhile, protonated SBs turned down quickly toward the EC side and preferred the EC-pointing configuration in simulations in which D96 was protonated. However, some notable differences between simulations starting in N’ and those of the M-intermediate structures occurred. For instance, when D96 was deprotonated a substantial CP-pointing population was observed only in the simulations starting from the 1F4Z M2 intermediate structure, and not in those starting from the 1KG8 and 1C8S structures, even though the cytoplasmic proton uptake pathway became hydrated in all the simulations (**Figure S5**). Similarly when D212 was protonated, the CP-pointing configuration predominated only in the simulations that started in the 1F4Z M2 intermediate structure. The effects of the deprotonating D96 and protonating D212 on the stability of the CP-pointing configuration in the 1F4Z M2 intermediate structure therefore mimicked their effects on the 1P8U N’ intermediate structure whereas structural features present in the other two M intermediate appear to favor the EC-pointing configurations. It is not clear what specific structural differences between 1F4Z and the other two M intermediate structures might be responsible for the different SB dynamics. One speculation is that the number of the initial water molecules in the D96-K216 cavity may play a role in this process. The number of the crystal water molecules in 1P8U and 1F4Z are both greater than those in 1KG8 and 1C8S. The crystal water molecules create a polar environment above the SB (the CP side), which could balance the polar attraction from below the SB, e.g. in the case that D212 is protonated.

## Discussion

Our simulations show that the protonated SB isomerizes from a CP-pointing configuration to an EC-pointing configuration on a pico to nanosecond time range. We interpret this to mean that the isomerization of the SB during switch II is therefore a process characterized by a low activation energy barrier. Two structural factors make major contributions to this energy landscape. The first is the protonation of the SB itself. Whereas the deprotonated form of the CP-pointing SB appears stable in nearly all cases, the protonated form rapidly isomerizes to an EC-pointing configuration. This observation is consistent with earlier computational studies that indicated the protonation of the SB significantly reduces the energy barrier for retinal isomerization [56] and with experimental observation that switch I, i.e. re-orientation of the deprotonated SB from EC-pointing to CP-pointing in the M state is a slow process occurring on a millisecond time scale [33], [57]. In addition to SB protonation, we observe that the protonation state of D212 plays a crucial role in the SB component of switch II. In this case, protonation of D212 can either abolish or significantly delay the switch. D212 is believed to remain deprotonated throughout the photocycle of BR and may be only transiently protonated by D85 during the O-to-BR transition. We suggest that the electrostatic attraction between the protonated SB and the deprotonated D212 may be a major contributor to the low activation energy barrier for the SB component of switch II during the M, N and O intermediates, which helps to favor a EC-pointing configuration. When D85 is deprotonated during the ground state, K, and L intermediates it further contributes to the stability of the EC-pointing state [58]. To quantify the electrostatic interactions from D212, we computed the Columbic interactions between the protonated SB and D212 for the nine trajectories in **Figure 2** panels b-d. As shown in **Figure S6**, the electrostatic interactions between the protonated SB and D212 reaches below −10 kcal/mol when D212 is deprotonated, whereas it is near zero or positive when D212 is protonated.

Furthermore, our simulations indicate that the dynamics and kinetics of the isomerization process of the reprotonated SB can be influenced by changes of the hydration of its local environment. In the normal photocycle of the wild type BR, the protonation state of D96 seems to also be a major factor. During the rise of the N state, when D96 deprotonates and the SB reprotonates, a water channel forms, connecting the SB with the cytoplasm. This creates a hydrated polar environment above the SB. When the polar interactions between water and the protonated SB out-weighs the attraction from D212, the water molecules in the channel may act as a diffuse counter-ion temporarily stabilizing the protonated SB in a CP-pointing configuration. Whether the CP-pointing protonated SB in the water channel may lead to proton backflow depends on the pKa of the SB. Although there is no experimental data on the pKa value of the SB after its reprotonation in the N state, it is around 13.3 in the ground state [59], [60] and it was suggested that this value may have already been restored after switch I is completed [33]. When the N state decays to the O state, D96 reprotonates and the cytoplasmic path recloses. The D96-K216 cavity above the SB returns back to hydrophobic and the attractive force from D212 finally settles down the protonated SB toward the EC side. This prepares the SB for a next cycle that is to release a proton to the EC side, ensuring the directionality of proton transport.

Finally, during the photocycle of BR the retinal and SB are involved in at least six molecular reactions central to ion pumping, including two isomerization (I) reactions of the retinal C13 = C14 bond, two proton transfer (T) reactions on the SB, two switches in orientation (EC vs. CP pointing) (S) of the SB (essentially the isomerization reactions of the C15 = NZ bond). Our simulations suggest that the thermal isomerization of retinal from *13-cis* back to the *all-trans* occurs after the SB switch II, as the retinal remained *13-cis* in all of the simulations. Also, as described in the Introduction section, experimental data show that during the wild-type photocycle the retinal is *13-cis* in the N intermediate and that it re-isomerizes back to *all-trans* only in the O state on a millisecond time scale [35], [36]. The activation energy barrier was computed to be ∼14 kcal/mol when D85 was modeled in a protonated form, mimicking its state during the N-to-O transition [21]. From the standpoint of the retinal, this contrasts to the conceptual isomerization-switch-transfer (IST) model proposed by Haupts et al. [61], in which the isomerization of the retinal was assumed to always precede the switch of the accessibility of the SB. Specifically, in switch II, the retinal thermal isomerization from 13-*cis* to all-*trans* occurs after reprotonation of the SB from D96 and is followed by SB’s isomerization from CP-pointing to EC-pointing during the N-to-O transition. The sequence of molecular events for the chromophore during the complete wild-type BR photocycle was thus proposed to be I/T/S/T/I/S. By contrast, our results lead us to propose that the sequence of these six molecular reactions is likely I/T/S/T/S/I. The I/T/S/T/I/S model would require the existence of a protonated all-*trans*, 15-*syn* configuration (CP-pointing), a state that has not been observed experimentally. Conversely, our model is consistent with the detection of both the *13-cis*,*15-syn* configuration (EC-pointing) and the *all-trans*, *15-anti* configuration (the EC-pointing ground state) in the dark-adapted BR [62], [63] and in the acid-blue form [64].

## Supporting Information

Figure S1 **Time development of the isomeric state of the protonated SB in solution.** The SB C15 = N double bond is in continuous interconversion between the *anti*- and *syn*- isomers, but with the *anti*-isomer being the predominant.
(TIF)

Figure S2 **Time development of the dihedral angles C14-C15 = NZ-CE (color symbols) and C12-C13 = C14-C15 (black symbols) in the simulations starting from the M1 structure (1KG8).** a) The SB was un-protonated and D96 was protonated, mimicking the M state; b) The SB was protonated and D96 was deprotonated, mimicking the rising of the N state; c) The SB was protonated and D96 was protonated, mimicking the decay of the N state; d) The SB, D96 and D212 were protonated. The black symbols in each of the plots depict the isomeric state of the retinal C13 = C14 double bond, which remained its initial *13-cis* configuration in all simulations.
(TIFF)

Figure S3 **Time development of the dihedral angles C14-C15 = NZ-CE (color symbols) and C12-C13 = C14-C15 (black symbols) in the simulations starting from the M2 structure (1F4Z).** a) The SB was un-protonated and D96 was protonated, mimicking the M state; b) The SB was protonated and D96 was deprotonated, mimicking the rising of the N state; c) The SB was protonated and D96 was protonated, mimicking the decay of the N state; d) The SB, D96 and D212 were protonated. The black symbols in each of the plots depict the isomeric state of the retinal C13 = C14 double bond, which remained its initial *13-cis* configuration in all simulations.
(TIFF)

Figure S4 **Time development of the dihedral angles C14-C15 = NZ-CE (color symbols) and C12-C13 = C14-C15 (black symbols) in the simulations starting from the Mn structure (1C8S).** a) The SB was un-protonated and D96 was protonated, mimicking the M state; b) The SB was protonated and D96 was deprotonated, mimicking the rising of the N state; c) The SB was protonated and D96 was protonated, mimicking the decay of the N state; d) The SB, D96 and D212 were protonated. The black symbols in each of the plots depict the isomeric state of the retinal C13 = C14 double bond, which remained its initial *13-cis* configuration in all simulations.
(TIFF)

Figure S5 **Time development of the number of the water molecules in the D96-K216 cavity, starting from four crystal structures 1p8u, 1kg8 1f4z and 1c8s.** For each crystal structure, nine simulations were shown in three columns. Left column: three simulations with un-protonated SB and protonated D96; middle column: three simulations with protonated SB and deprotonated D96; right column: three simulations with protonated SB and D96.
(TIF)

Figure S6 **Coulombic potential between Schiff-base NH and D212 COO^-^ groups for the 9 molecular dynamics trajectories shown in** **Figure 2** **.** The Coulombic potential between the Schiff-base NH and D212 COO^-^ groups for the 9 trajectories in Figure 2 (panels b to d) are shown. The colors correspond in this figure correspond to those used in the main text Figure 2. Briefly, (a) The SB was protonated and D96 was deprotonated, mimicking the rising of the N state; (b) The SB was protonated and D96 was protonated, mimicking the decay of the N state; (c) The SB, D96 and D212 were protonated. These show that when the Schiff base is pointing towards D212 that the electrostatic interactions reach below −10 kcal/mol, while when the Schiff-base points away from D212, or D212 is protonated, the electrostatic potential is around 0 or positive.
(TIF)

Table S1 Properties of the four crystal structures used in our simulations.
(DOC)

Table S2 Result summary of 48 MD simulations, complement to Fig.5 in the main text.
(DOC)
