# Supplementary figures and images for: Schiff Base Switch II Precedes the Retinal Thermal Isomerization in the Photocycle of Bacteriorhodopsin
Source: PLoS One. 2013 Jul 29;8(7):e69882. doi: 10.1371/journal.pone.0069882 (PMC3726731; doi:10.1371/journal.pone.0069882)

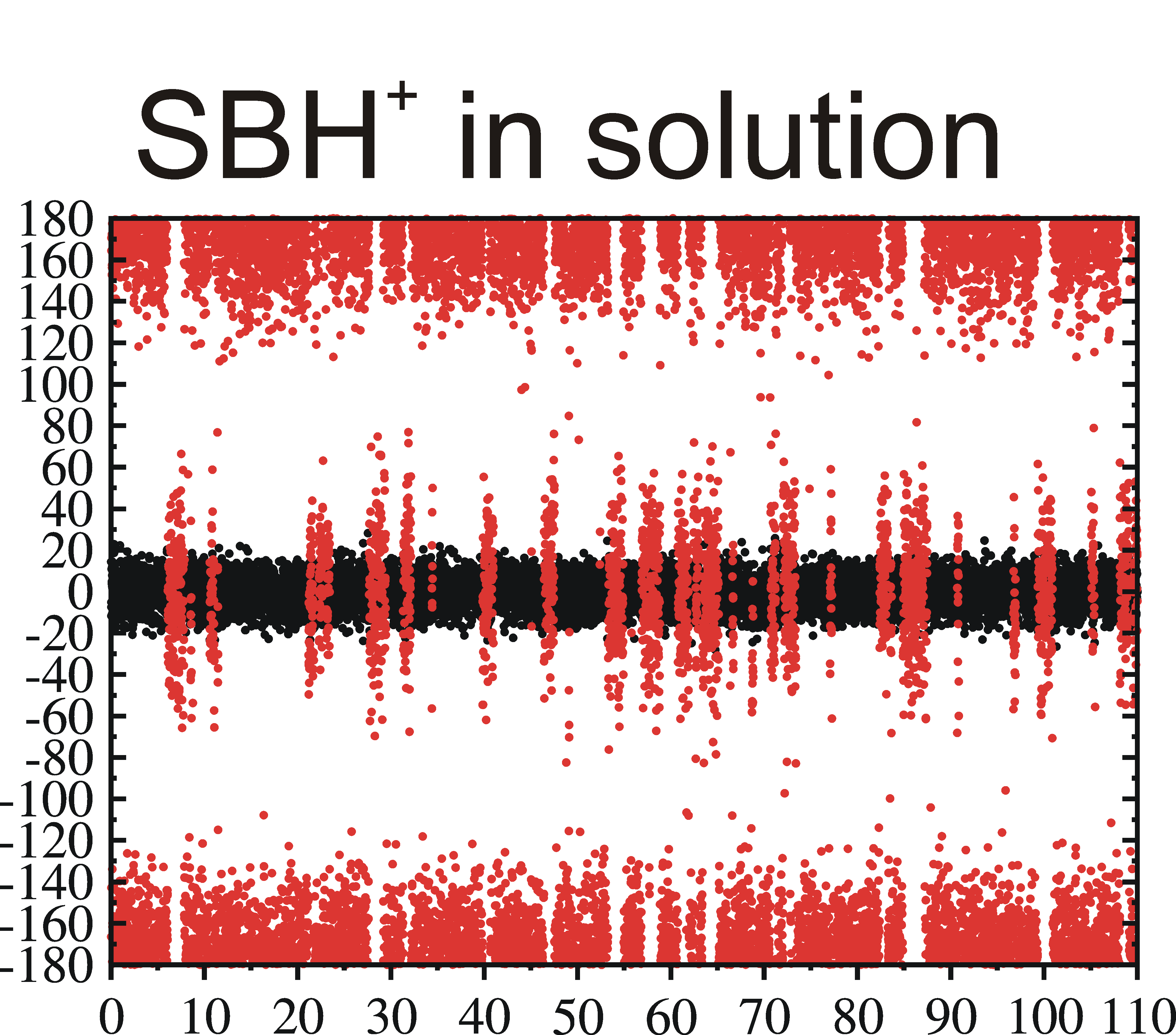

Supplement: Figure S1 — Time development of the isomeric state of the protonated SB in solution. The SB C15 = N double bond is in continuous interconversion between the anti- and syn- isomers, but with the anti-isomer being the predominant. (TIF) [file pone.0069882.s001.tif]

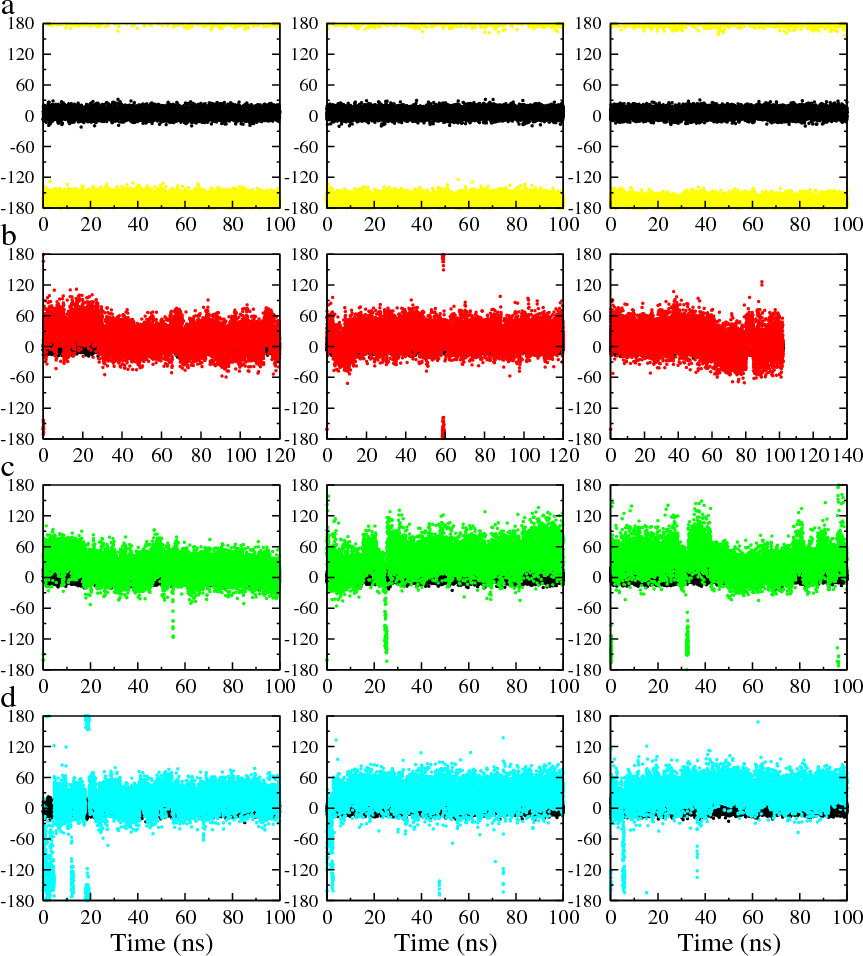

Supplement: Figure S2 — Time development of the dihedral angles C14-C15 = NZ-CE (color symbols) and C12-C13 = C14-C15 (black symbols) in the simulations starting from the M1 structure (1KG8). a) The SB was un-protonated and D96 was protonated, mimicking the M state; b) The SB was protonated and D96 was deprotonated, mimicking the rising of the N state; c) The SB was protonated and D96 was protonated, mimicking the decay of the N state; d) The SB, D96 and D212 were protonated. The black symbols in each of the plots depict the isomeric state of the retinal C13 = C14 double bond, which remained its initial 13-cis configuration in all simulations. (TIFF) [file pone.0069882.s002.tiff]

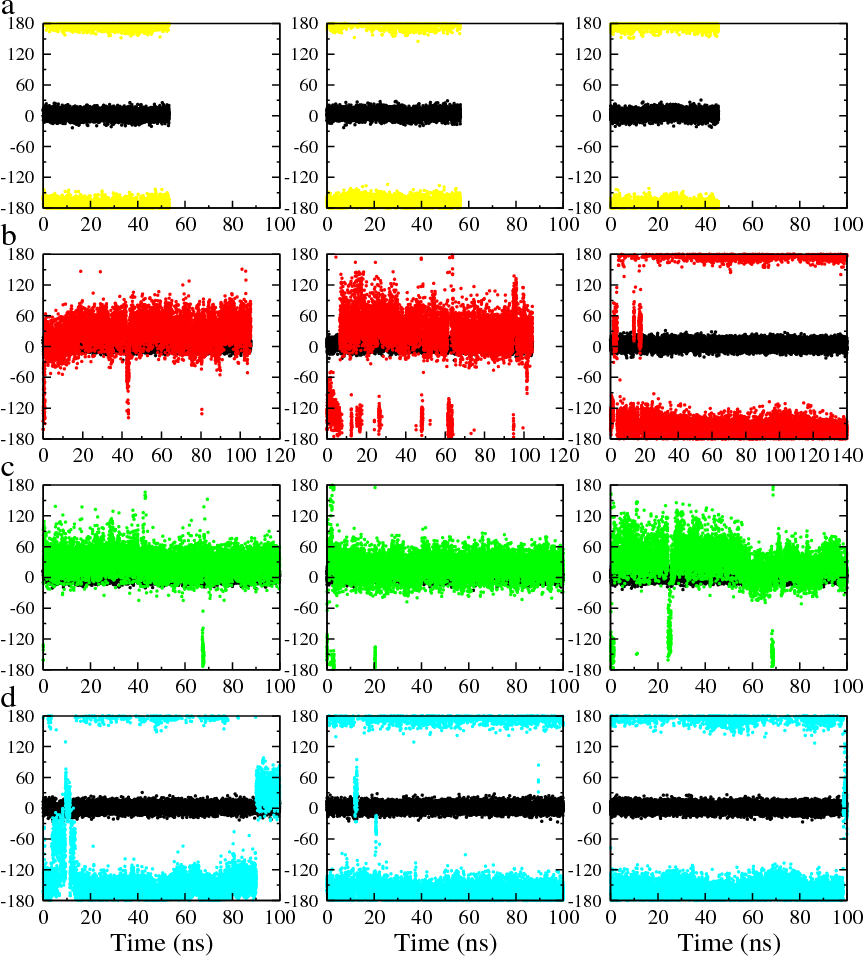

Supplement: Figure S3 — Time development of the dihedral angles C14-C15 = NZ-CE (color symbols) and C12-C13 = C14-C15 (black symbols) in the simulations starting from the M2 structure (1F4Z). a) The SB was un-protonated and D96 was protonated, mimicking the M state; b) The SB was protonated and D96 was deprotonated, mimicking the rising of the N state; c) The SB was protonated and D96 was protonated, mimicking the decay of the N state; d) The SB, D96 and D212 were protonated. The black symbols in each of the plots depict the isomeric state of the retinal C13 = C14 double bond, which remained its initial 13-cis configuration in all simulations. (TIFF) [file pone.0069882.s003.tiff]

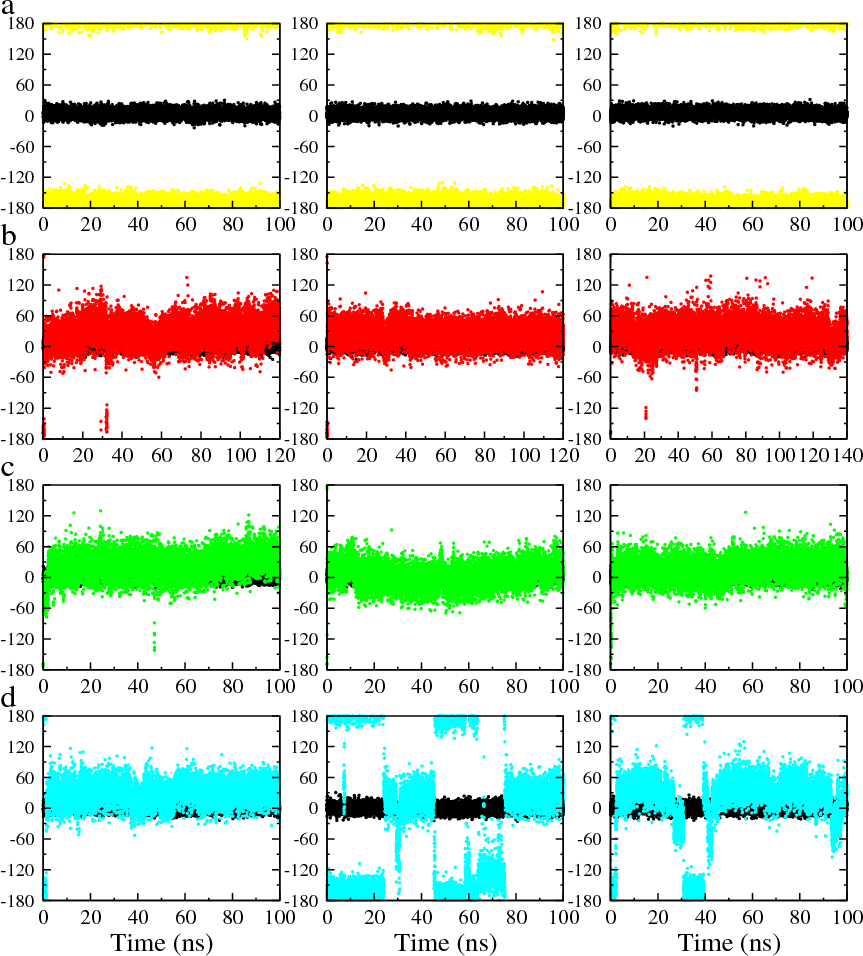

Supplement: Figure S4 — Time development of the dihedral angles C14-C15 = NZ-CE (color symbols) and C12-C13 = C14-C15 (black symbols) in the simulations starting from the Mn structure (1C8S). a) The SB was un-protonated and D96 was protonated, mimicking the M state; b) The SB was protonated and D96 was deprotonated, mimicking the rising of the N state; c) The SB was protonated and D96 was protonated, mimicking the decay of the N state; d) The SB, D96 and D212 were protonated. The black symbols in each of the plots depict the isomeric state of the retinal C13 = C14 double bond, which remained its initial 13-cis configuration in all simulations. (TIFF) [file pone.0069882.s004.tiff]

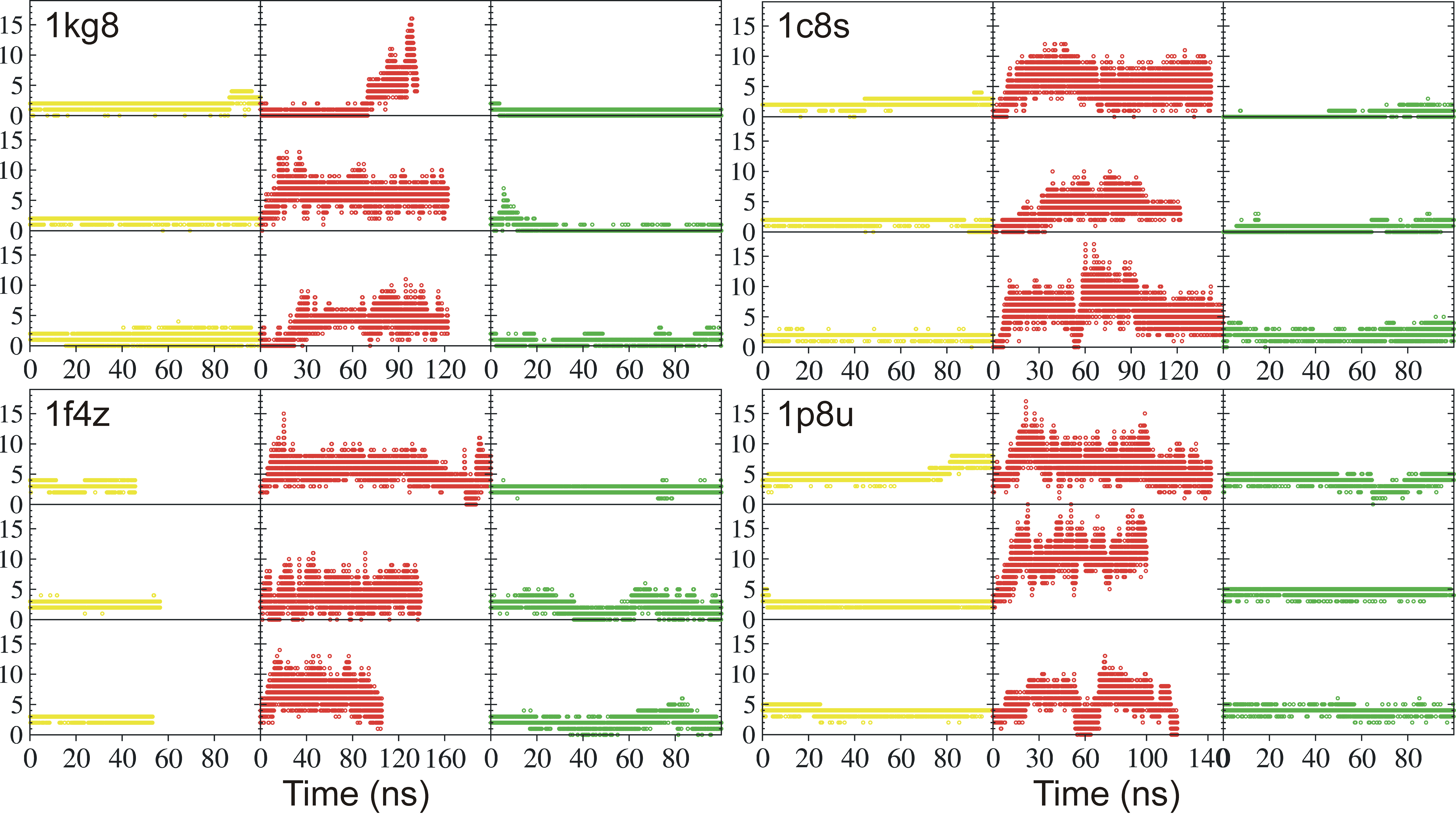

Supplement: Figure S5 — Time development of the number of the water molecules in the D96-K216 cavity, starting from four crystal structures 1p8u, 1kg8 1f4z and 1c8s. For each crystal structure, nine simulations were shown in three columns. Left column: three simulations with un-protonated SB and protonated D96; middle column: three simulations with protonated SB and deprotonated D96; right column: three simulations with protonated SB and D96. (TIF) [file pone.0069882.s005.tif]

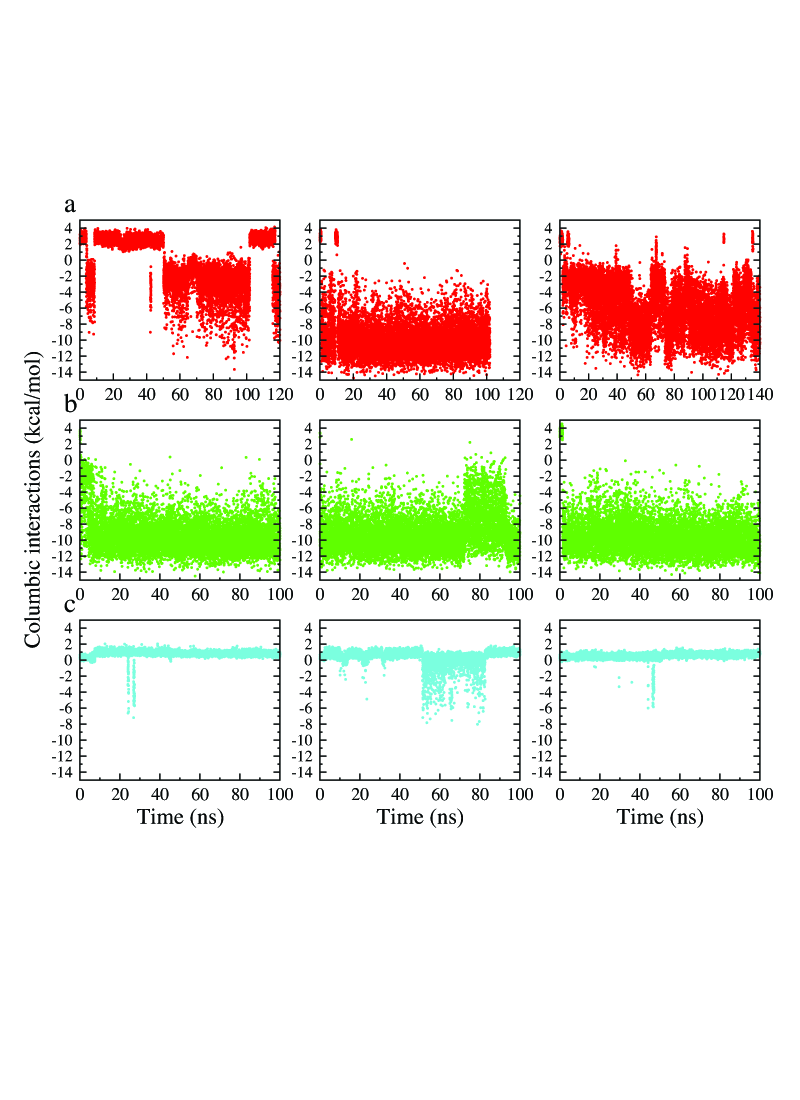

Supplement: Figure S6 — Coulombic potential between Schiff-base NH and D212 COO- groups for the 9 molecular dynamics trajectories shown in Figure 2 . The Coulombic potential between the Schiff-base NH and D212 COO- groups for the 9 trajectories in Figure 2 (panels b to d) are shown. The colors correspond in this figure correspond to those used in the main text Figure 2. Briefly, (a) The SB was protonated and D96 was deprotonated, mimicking the rising of the N state; (b) The SB was protonated and D96 was protonated, mimicking the decay of the N state; (c) The SB, D96 and D212 were protonated. These show that when the Schiff base is pointing towards D212 that the electrostatic interactions reach below −10 kcal/mol, while when the Schiff-base points away from D212, or D212 is protonated, the electrostatic potential is around 0 or positive. (TIF) [file pone.0069882.s006.tif]
