# Supplementary material for: Schiff Base Switch II Precedes the Retinal Thermal Isomerization in the Photocycle of Bacteriorhodopsin
Source: PLoS One. 2013 Jul 29;8(7):e69882. doi: 10.1371/journal.pone.0069882 (PMC3726731; doi:10.1371/journal.pone.0069882)
Supplement: Table S1 — Properties of the four crystal structures used in our simulations. (DOC) [file pone.0069882.s007.doc]

**Table S1**. Properties of the four crystal structures used in our simulations.

| PDB | state | sample | Resolution  (Å) | residues solved in the structure | number of water in the D96-K216 cavity^a^ | D85:OD1-D212:OD2 distance (Å) | D212:OD2-NZ distance (Å) |
| --- | --- | --- | --- | --- | --- | --- | --- |
| 1KG8 | M1 | Wild type | 2.0 | 5-155,167-231 | 2 | 4.84 | 4.44 |
| 1F4Z | M2 | E204Q | 1.8 | 5-156,162-231 | 3 | 4.44 | 4.10 |
| 1C8S | Mn | D96N | 2.0 | 5-153,176-222 | 2 | 4.96 | 3.78 |
| 1P8U | N' | V49A | 1.62 | 5-156,162-231 | 4 | 4.40 | 3.99 |

^a^: WAT501 in 1F4Z and WAT504 in 1P8U were excluded by the definition of the D96-K216 cavity sphere used in this work.
