# Supplementary material for: Schiff Base Switch II Precedes the Retinal Thermal Isomerization in the Photocycle of Bacteriorhodopsin
Source: PLoS One. 2013 Jul 29;8(7):e69882. doi: 10.1371/journal.pone.0069882 (PMC3726731; doi:10.1371/journal.pone.0069882)
Supplement: Table S2 — Result summary of 48 MD simulations, complement to Fig.5 in the main text. (DOC) [file pone.0069882.s008.doc]

**Table S2**. Result summary of 48 MD simulations, complement to **Fig.5** in the main text.

| Simulation ^a^ | Protonation states ^b^ | *15-syn* % | *15-anti* % | Number of ***different*** water molecules that accessed the D96-K216 cavity | Trajectory length (ns) |
| --- | --- | --- | --- | --- | --- |
| P8U-a-1 | SB^0^, D96H^0^, D212^-^ | 0 | 100 | 6 | 102.02 |
| P8U-a-2 | SB0, D96H^0^, D212^-^ | 0 | 100 | 5 | 102.02 |
| P8U-a-3 | SB0, D96H^0^, D212^-^ | 0 | 100 | 8 | 102.02 |
| P8U-b-1 | SBH^+^, D96^-^, D212^-^ | 49.99 | 48.48 | 129 | 120.02 |
| P8U-b-2 | SBH^+^, D96^-^, D212^-^ | 96.66 | 1.87 | 374 | 102.02 |
| P8U-b-3 | SBH^+^, D96^-^, D212^-^ | 92.06 | 2.74 | 443 | 142.02 |
| P8U-c-1 | SBH^+^, D96H^0^, D212^-^ | 97.24 | 0.15 | 14 | 102.02 |
| P8U-c-2 | SBH^+^, D96H^0^, D212^-^ | 94.42 | 0.06 | 5 | 102.02 |
| P8U-c-3 | SBH^+^, D96H^0^, D212^-^ | 97.38 | 1.07 | 5 | 102.02 |
| P8U-d-1 | SBH^+^, D96H^0^, D212H^0^ | 0.79 | 97.57 | 5 | 102.02 |
| P8U-d-2 | SBH^+^, D96H^0^, D212H^0^ | 35.44 | 60.65 | 21 | 102.02 |
| P8U-d-3 | SBH^+^, D96H^0^, D212H^0^ | 0.81 | 98.72 | 5 | 102.02 |
| KG8-a-1 | SB^0^, D96H^0^, D212^-^ | 0 | 100 | 4 | 102.02 |
| KG8-a-2 | SB0, D96H^0^, D212^-^ | 0 | 100 | 2 | 102.02 |
| KG8-a-3 | SB0, D96H^0^, D212^-^ | 0 | 100 | 4 | 102.02 |
| KG8-b-1 | SBH^+^, D96^-^, D212^-^ | 94.57 | 0.14 | 331 | 122.02 |
| KG8-b-2 | SBH^+^, D96^-^, D212^-^ | 97.16 | 0.64 | 774 | 122.02 |
| KG8-b-3 | SBH^+^, D96^-^, D212^-^ | 97.13 | 0.04 | 361 | 102.02 |
| KG8-c-1 | SBH^+^, D96H^0^, D212^-^ | 96.75 | 0.04 | 13 | 102.02 |
| KG8-c-2 | SBH^+^, D96H^0^, D212^-^ | 81.31 | 0.55 | 25 | 102.02 |
| KG8-c-3 | SBH^+^, D96H^0^, D212^-^ | 84.22 | 1.21 | 2 | 102.02 |
| KG8-d-1 | SBH^+^, D96H^0^, D212H^0^ | 92.72 | 5.27 | 2 | 102.02 |
| KG8-d-2 | SBH^+^, D96H^0^, D212H^0^ | 94.13 | 0.31 | 3 | 102.02 |
| KG8-d-3 | SBH^+^, D96H^0^, D212H^0^ | 90.80 | 0.65 | 2 | 102.02 |
| F4Z-a-1 | SB^0^, D96H^0^, D212^-^ | 0 | 100 | 3 | 53.23 |
| F4Z-a-2 | SB0, D96H^0^, D212^-^ | 0 | 100 | 5 | 56.47 |
| F4Z-a-3 | SB0, D96H^0^, D212^-^ | 0 | 100 | 5 | 45.64 |
| F4Z-b-1 | SBH^+^, D96^-^, D212^-^ | 88.13 | 0.40 | 442 | 105.34 |
| F4Z-b-2 | SBH^+^, D96^-^, D212^-^ | 75.21 | 9.49 | 467 | 104.33 |
| F4Z-b-3 | SBH^+^, D96^-^, D212^-^ | 2.03 | 97.24 | 627 | 184.45 |
| F4Z-c-1 | SBH^+^, D96H^0^, D212^-^ | 84.51 | 2.31 | 8 | 102.02 |
| F4Z-c-2 | SBH^+^, D96H^0^, D212^-^ | 92.80 | 0.77 | 13 | 102.02 |
| F4Z-c-3 | SBH^+^, D96H^0^, D212^-^ | 96.04 | 1.07 | 4 | 102.02 |
| F4Z-d-1 | SBH^+^, D96H^0^, D212H^0^ | 20.76 | 72.39 | 4 | 102.02 |
| F4Z-d-2 | SBH^+^, D96H^0^, D212H^0^ | 1.13 | 98.41 | 3 | 102.02 |
| F4Z-d-3 | SBH^+^, D96H^0^, D212H^0^ | 11.66 | 87.53 | 23 | 102.02 |
| C8S-a-1 | SB^0^, D96H^0^, D212^-^ | 0 | 100 | 3 | 102.02 |
| C8S-a-2 | SB0, D96H^0^, D212^-^ | 0 | 100 | 2 | 102.02 |
| C8S-a-3 | SB0, D96H^0^, D212^-^ | 0 | 100 | 4 | 102.02 |
| C8S-b-1 | SBH^+^, D96^-^, D212^-^ | 88.96 | 0.46 | 1299 | 142.02 |
| C8S-b-2 | SBH^+^, D96^-^, D212^-^ | 96.74 | 0.16 | 147 | 122.02 |
| C8S-b-3 | SBH^+^, D96^-^, D212^-^ | 93.48 | 0.13 | 479 | 142.02 |
| C8S-c-1 | SBH^+^, D96H^0^, D212^-^ | 93.60 | 0.10 | 7 | 102.02 |
| C8S-c-2 | SBH^+^, D96H^0^, D212^-^ | 99.17 | 0.05 | 13 | 102.02 |
| C8S-c-3 | SBH^+^, D96H^0^, D212^-^ | 97.68 | 0.12 | 5 | 102.02 |
| C8S-d-1 | SBH^+^, D96H^0^, D212H^0^ | 90.94 | 1.23 | 5 | 102.02 |
| C8S-d-2 | SBH^+^, D96H^0^, D212H^0^ | 45.23 | 49.14 | 2 | 102.02 |
| C8S-d-3 | SBH^+^, D96H^0^, D212H^0^ | 79.16 | 10.40 | 7 | 102.02 |

^a^: The name of each simulation began with the first three letters of the crystal structure PDB code and followed by letter a, b, c or d, then number 1, 2 or 3. For example, name kg8-a-1 indicates that this was the first simulation that started from the crystal structure of 1KG8 and the protonation states of SB and residues D96 and D212 correspond to panel a in **Figure 2**. The rows are colored corresponding to the colors of the symbols in panels a, b, c and d in **Figure 2**.

^b^: SB^0^, unprotonated Schiff base; SBH^+^, protonated Schiff base; D96H^0^, protonated D96; D96^-^ deprotonated D96; D212^-^, deprotonated D212; D212H^0^, protonated D212; D85 and D115 were protonated in all simulations.
